# WARCProcessor: An Integrative Tool for Building and Management of Web Spam Corpora

**DOI:** 10.3390/s18010016

**Published:** 2017-12-22

**Authors:** Miguel Callón, Jorge Fdez-Glez, David Ruano-Ordás, Rosalía Laza, Reyes Pavón, Florentino Fdez-Riverola, Jose Ramón Méndez

**Affiliations:** 1 ESEI: Higher Technical School of Computer Engineering, University of Vigo, 32004 Ourense, Spain; miguel.callon.alvarez@gmail.com (M.C.); drordas@uvigo.es (D.R.-O.); rlaza@uvigo.es (R.L.); pavon@uvigo.es (R.P.); moncho.mendez@uvigo.es (J.R.M.); 2 CINBIO: Biomedical Research Centre, Campus Universitario Lagoas-Marcosende, 36310 Vigo, Spain; 3 CITI: Centre for Research Transference and Innovation, Avda. Galicia 2, Parque Tecnolóxico, San Cibrao das Viñas, 32900 Ourense, Spain; jfgonzalez3@gmail.com

**Keywords:** web spam research, corpus generation and maintenance, multiple data sources, WARC format 1.0

## Abstract

In this work we present the design and implementation of WARCProcessor, a novel multiplatform integrative tool aimed to build scientific datasets to facilitate experimentation in web spam research. The developed application allows the user to specify multiple criteria that change the way in which new corpora are generated whilst reducing the number of repetitive and error prone tasks related with existing corpus maintenance. For this goal, WARCProcessor supports up to six commonly used data sources for web spam research, being able to store output corpus in standard WARC format together with complementary metadata files. Additionally, the application facilitates the automatic and concurrent download of web sites from Internet, giving the possibility of configuring the deep of the links to be followed as well as the behaviour when redirected URLs appear. WARCProcessor supports both an interactive GUI interface and a command line utility for being executed in background.

## 1. Introduction

Nowadays, the World Wide Web (WWW) has become an essential source of information in almost every area of knowledge. This situation have led to the critical need for powerful search engines able to instantly provide access to the required information. Those search engines supply ordered lists containing the best results related to the user query based on intelligent page ranking algorithms. In this context, the ranking of pages represents a key component determining how users browse the web, motivating the development of a large number of techniques aimed at improving the accuracy of the ranking process [1,2,3]. As a result, different advanced search engine-positioning methods like Search Engine Optimization (SEO) or Search Engine Marketing (SEM) were recently implemented, being widely used.

In such a situation, given the economic importance of occupying prominent positions in rankings, different illegal methods were also developed for specifically deceiving the ranking algorithms internally used by common search engines [4]. Such pernicious techniques are generally known as web spamming practices or spamdexing procedures. At present, web spamming activities have a decisive detrimental effect on the quality of search results, causing a considerable waste of users’ time and a decrease in the trust bestowed upon major search engine providers [5]. Additionally, recent studies indicate that the amount of web spam is dramatically increasing instead of reducing [6,7]. In this context, the effective elimination of web spam entries from search results represents an essential unresolved issue.

However, WWW is not only the greatest available repository of digital information, but also the largest communication platform. This characteristic has inspired the construction of web sites and discussion forums (e.g., blogs, forums, social networking, media sharing sites, wikis, e-commerce and online reviews) where people present their personal experiences, emotions, attitudes and feelings regarding not only products and services, but also political and economic issues in our society. The open nature of WWW enables millions of users to quickly share information and opinions instead of passively viewing contents. In such a situation, the widespread sharing and utilization of online opinions in web sites has increased spam content at enormous rate, mainly due to the influence of these comments for individuals and organizations when purchasing and/or making business decisions [8,9]. Besides spamdexing, spam 2.0 is also another well-known form of spam affecting WWW Internet service [10,11,12,13], being usually connected with the usage of fake user profiles or Sybil accounts [13,14]. Despite the fact spam 2.0 and spamdexing could be connected by the influence of social activities in search engines [13], there are key differences between spamdexing and other forms of spam such as: (i) information granularity and (ii) the importance of user profiles. While data granularity in spamdexing is represented by an entire website or a given web page, other forms of spam require the analysis of small parts of a web page such as a comment, a wall post or an image label. In the same line, user profile information is not available for spamdexing purposes, while it becomes relevant in other forms of WWW spam [12] or even target problem [13,14,15]. As it was to be expected, the overwhelming amount of spam is seriously degrading the quality of the information available on WWW, imposing at the same time new challenges for the entire web community [10,11].

This situation has motivated a growing interest of the research community for (i) studying this novel form of web spam, mainly related with the occurrence of the Web 2.0; (ii) the proposal of novel and more accurate algorithms and techniques for its effective detection and; (iii) the analysis of new challenges imposed to the existing information retrieval systems. In this context, several research works show how web spam usually appears in sophisticated forms using several tricks to mislead search engines for assigning higher ranks to fake sites [16,17,18,19,20,21]. Complementarily, different works show how to assess the problem of spam in social web sites developing specific solutions [9,22,23,24,25,26].

In any case, the detection of spam on the web has been mainly addressed as a classification problem (i.e., spam vs. legitimate content) [21]. Although every approach has its own characteristics and limitations, all of them require the availability, management and use of large amount of electronic collections of previously classified web sites (known as corpora) to correctly train and evaluate the proposed approaches. Therefore, one of the biggest challenges today is to obtain reliable labeled data with the aim of both facilitating the comparison between existing alternatives and boosting the accuracy of novel anti-spam methods [8,27].

In this regard, there are only a few web spam corpora publicly available that can be successfully used to train, test, compare and rank existing and novel approaches for effective web spam detection and filtering. Moreover, most of the available alternatives are outdated and distributed in different incompatible formats [8,9,11,18,19,23,26,28,29,30,31,32]. This situation forces research teams to always carry out a previous compulsory task of data preparation and preprocessing [29], which in web spam-filtering domain habitually becomes hard, costly, time consuming and prone to error.

The Web 2.0 concept was recently introduced to characterize a group of web applications able to maximize the interaction of their users by enabling them to contribute, collaborate and edit site-contents. The popularization of these trends caused a growth in the frequency of website content changes (currently changes take place second by second). Moreover, website contents could become unavailable due to the temporal failures, the company ceasing operation, or when websites are closed by authorities. A consequence of such a situation is that the verification of previous experimental findings becomes complicated (the original dataset cannot be recovered), hampering the scientific progress and the consolidation of valid knowledge [33]. In this context, there is an obvious need to have a flexible tool to support building web spam corpora through the combination of information from multiple data sources, downloading all contents from compiled websites and storing a local copy of them using standard formats (WARC, https://iipc.github.io/ warc-specifications/specifications/warc-format/warc-1.0/).

Such kind of tools are somewhat common in different application areas also needing to manage large collections of electronic documents. Examples of these complementary applications from other related domains are the following: EXMARaLDA corpus manager [34], designed for working with oral corpora and consisting of a transcription and annotation tool, a tool for managing corpora and a query and analysis tool; WebBootCaT [35], a web service for quickly producing corpora for specialist areas, which can be further explored with an accompanying corpus query tool (Sketch Engine); Genetic Programming for Crawling (GP4C) framework to generate score functions that produce accurate rankings of pages regarding their probabilities of having been modified [36].

However, to the best of our knowledge, there is not an available tool specifically focused on giving support to all the singularities that characterize the web spam-filtering domain. Among other relevant functionalities, researchers essentially need to easily manage web spam corpora (containing already classified spam/legitimate entries) from different and configurable data sources (e.g., blacklists, previously downloaded corpus, etc.), being usually stored using diverse formats. Apart from this basic functionality, there is also the common need of enabling the user to automatically create new corpora based on different selection criteria (e.g., size of the new corpus, distribution of spam/legitimate contents, use of a target language, etc.) with the goal of establishing a reproducible experimental framework for further research.

On the basis of the above this work presents WARCProcessor, a platform-independent integrative tool providing specific support to scientists to build appropriate datasets in the field of web spam, avoiding time consuming tasks, and storing these metadata for future comparisons in an interoperable format. In detail, our developed application is specialized in the generation of curated corpus for training and validation purposes, widely supports the standard WARC format and easily allows the execution of user workflows using both GUI and command line interfaces. Moreover, WARCProcessor provides transparent deployment of new software versions, and can be executed on any computer without the need of downloading and installing additional packages. The tool is open source, being freely available to the scientific community from its web site (http://sing-group.org/warcprocessor).

While this section has motivated the work, the rest of the paper is organized as follows: Section 2 describes the available web spam corpora and discusses how previous researches have preprocessed them with the goal of identifying existing shortfalls; Section 3 details the system architecture and discusses the design principles that guide the current implementation. In order to globally assess the value of WARCProcessor; Section 4 introduces different usage scenarios reporting the execution times for the various operations carried out; Finally, Section 5 outlines main conclusions of the work and establishes future lines of development.

## 2. Available Corpora, Required Preprocessing and Verification Issues

Given the specific nature of the web spam domain, and with the aim of properly analyze key aspects related with the design and implementation of novel anti-spam approaches, this section details the publicly available corpora for web spam research, identifies the main shortcomings regarding their processing and discusses the importance of correctly verify experimental findings.

In relation with the existence of different ready-to-use repositories for validating novel methods and techniques to fight web spam, there are six publicly available corpora and two other alternatives to which access is restricted. Table 1 summarizes their most outstanding characteristics.

Regarding the corpora shown in Table 1, the uk2006 and uk2007 datasets were initially collected and later labeled by laboratory volunteers at the University of Milan. The first collection is formed by 77.9 million of web pages belonging to 11,402 hosts for UK domains, whilst the second is composed of 105,896,555 web pages belonging to 114,529 hosts. Later, the uk2011 dataset was derived from the uk2007 corpus containing 3767 web pages.

Alternatively, DC2010 includes a large collection of annotated web hosts labeled by the Hungarian Academy of Sciences, the Internet Memory Foundation and the L3S Research Center. The base data is composed of 23 million of web pages belonging to 99,000 hosts for the .eu domain crawled by the Internet Memory Foundation in early 2010.

For their part, the Webb spam 2006 (collected by Steve Webb) and Webb spam 2011 (collected by De Wang) corpora were generated starting from the Denial of Information Project at the Georgia Institute of Technology. It was the first time that a large scale and publicly available web spam corpora were created using a fully automated web spam collection method. In detail, the Webb spam 2011 corpus is formed by 330,000 web spam pages, making it more than two orders of magnitude larger than any of the previously available alternatives.

With a more precise goal, the clueweb 09 and 12 corpora were created to support specific research on information retrieval and related human language technologies, being used in several tracks of the annual Text REtrieval Conference (TREC) series. The first edition of the corpus consists of about 1 billion of web pages written in 10 different languages (500 million of web pages in English) that were collected from January to February, 2009. A second (updated) version of this corpus (clueweb 12) was collected between 10 February 2012 and 10 May 2012 and stores 733,019,372 web pages.0

In recent years, all these corpora were used in several ways by different researchers applying distinct preprocessing techniques but with the same goal: the comprehensive evaluation of novel strategies and approaches for fighting web spam. In this way, Silva and researchers worked with the uk2006 and uk2007 collections needing to remove all feature vectors with no assigned labels or labeled as undefined [19]. After this procedure, the authors obtained a specific subset containing 76.6% hosts labeled as legitimated and 23.4% hosts categorized as spam. Similarly, in [30] the authors generated a new subset from the uk2007 and uk2011 datasets based on different extracted features. In both cases, the authors chose only those pages currently active (up and running) and also manually labeled new Arabic spam pages (taking into account different content-based features) with the goal of complementing their initial corpus.

Complementarily, Han and Levenberg worked with the uk2007 corpus for developing an online incremental learning framework to detect web spam [37]. From their angle, a major problem with the selected corpus was that spam data are less common than legitimate entries. Consequently, they needed to consider a proper strategy to deal with unbalanced data during sample selection.

From a different perspective, in the work [20] the authors combined data from the uk2007 and Webb spam corpora with the goal of identifying novel properties to detect web spam. As these corpora only contain spam entries, the authors needed to manually add legitimate pages tagging their content from a Yahoo! dataset.

In relation with the spam happening in social network web sites, Wang and colleagues proposed SPADE [29], a framework specially focused on providing spam analytics and detection features. In their experimental protocol, the authors used different datasets, mapping and assembly of multiple processes, pre-filtering, feature selection techniques and classification approaches.

In parallel, Garzó and researchers studied how existing models could be successfully translated from English into another language and how language-dependent and independent methods could be combined [31]. The authors finally selected training and test data in different languages from the clueweb09 corpus.

Additionally, Erdélyi and colleagues presented a comprehensive evaluation of several features devised for web spam detection, carrying out different tests with uk2007, DC2010 and clueweb09 corpora [18]. To foster the research in the area, the authors made publicly available several feature sets and source code containing the temporal attributes of eight .uk crawl snapshots, including uk2007 together with the Web Spam Challenge features for the labeled part of clueweb09 corpus. On this occasion, the authors had to process different corpora stored in incompatible formats.

Finally, Samar and investigators analyzed and discussed the balance between reproducibility and representativeness when building test collections [32]. They focused their analysis on the Contextual Suggestion TREC track, for which in 2013 and 2014 it was possible to submit runs based on either Open Web or ClueWeb12, a static version of the web. The authors had to normalize URLs by removing their prefixes (e.g., www, http://, https://) with the goal of identifying those documents included in OpenWeb that are also found in the ClueWeb12 collection. In this case, the authors needed to map all the URLs found by Open Web systems to their ClueWeb12 ids (overlapping corpus).

Keeping all of the above in mind (i.e., existing available corpora and specific preprocessing needs), the following key features were identified as essential to implement a powerful yet flexible corpus management software helping to ensure reproducible research [38] and giving an adequate support to the specific requirements of web spam researchers: (i) integration of available information previously classified from different data sources (e.g., backlists, whitelists, existing corpora, etc.); (ii) easy labelling (i.e., spam or legitimate) of pages and/or domains; (iii) configurable conditions (e.g., percentage of spam/ham content, size of the corpus, language, etc.) for the generation of novel subsets; (iv) appropriate support for accessing data stored in different formats and (v) automatic detection of dead links.

The next section presents the main characteristics and architecture of WARCProcessor, a recent development able to (i) particularly address previously identified requirements and (ii) avoid the execution of repetitive and error prone tasks.

## 3. WARCProcessor: Main Workflow, System Architecture and Execution Modes

As previously mentioned, WARCProcessor implements a flexible multiplatform tool, which provides specific support for integrating up to six commonly used data sources (see Table 2) to assist in the building/maintenance of configurable new corpora used for research activities in the web spam domain. By simply specifying different data sources containing previously classified URLs (i.e., csvDS, arffDS or fileDS) and/or their associated HTML content (i.e., warcDS, warccsvDS and corpusDS), the user of WARCProcessor can easily configure and create new corpora for giving support to his particular experimental protocol. As a central contribution of this work, this section presents a general overview covering the workflow that guides the generation process of the output corpus in WARCProcessor. Moreover, it also discusses the layered design of the tool, its component architecture as well as some implementation details. Finally, we also document the two execution modes available in our WARCProcessor tool: the interactive GUI interface and the command line utility.

### 3.1. WARCProcessor Workflow

**Figure 1:**
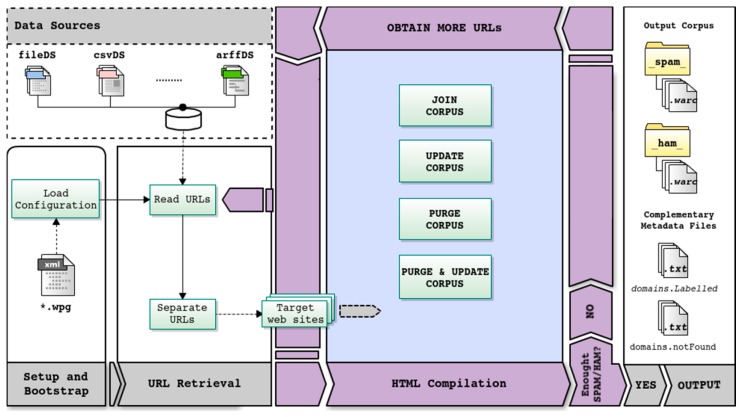
Main WARCProcessor workflow showing the supported use cases.

Although WARCProcessor provides multiple functionalities to the user through its numerous available options, there are four main use cases (i.e., tasks) for which it was specifically designed: (i) join corpus; (ii) update corpus; (iii) purge corpus and (iv) purge & update corpus. The central part of Figure 1 represents these essential use cases, surrounded by the common steps executed by WARCProcessor for generating the target corpus.

As Figure 1 details, given a previously available configuration (.wpg) file containing the specific user requirements for a given task, the generation process is structured into three sequential phases: (i) process setup and bootstrap; (ii) retrieval of URLs from data sources and (iii) content compilation from target web sites. During the process setup and bootstrap stage, WARCProcessor initializes its own data structures to handle those preconfigured input data sources and loads different user configuration details. Next, the URL retrieval stage is in charge of accessing the available data sources with the goal of (i) obtaining the required number of web sites comprising the final corpus (established by the user) and (ii) initially guaranteeing that the desired percentage rates of spam/legitimate entries are met. As a result of the execution of this phase, a list of target web sites is generated for being further processed.

After the completion of the HTML compilation stage, it is possible that the number of web sites finally included in the resulting corpus remains insufficient (motivated, for example, by the non-availability of some online web sites). In such a situation, WARCProcessor will return to the second step (URL Retrieval) in order to continue processing more URLs from data sources (as many as are required to achieve the target number). This step is repeated until the needed number of web sites is collected or any URLs from data sources are analyzed.

When WARCProcessor successfully accomplishes the assigned user task, it generates an output folder containing the following elements (see the right part of Figure 1): (i) two subfolders (called _ham_ and _spam_, respectively) which store the WARC files containing the HTML content of each original URL (where each WARC file corresponds to a given web site); (ii) a text file called domains.Labelled including those URLs which were successfully retrieved and stored into their corresponding WARC files and (iii) a text file named domains.notFound identifying those URLs that were both inaccessible from download and which content was not found in any available data source. Additionally, it should be mentioned that during the whole process (and without taking account of the specific task executed) WARCProcessor avoids storing duplicate web sites in the output corpus.

**Figure 2:**
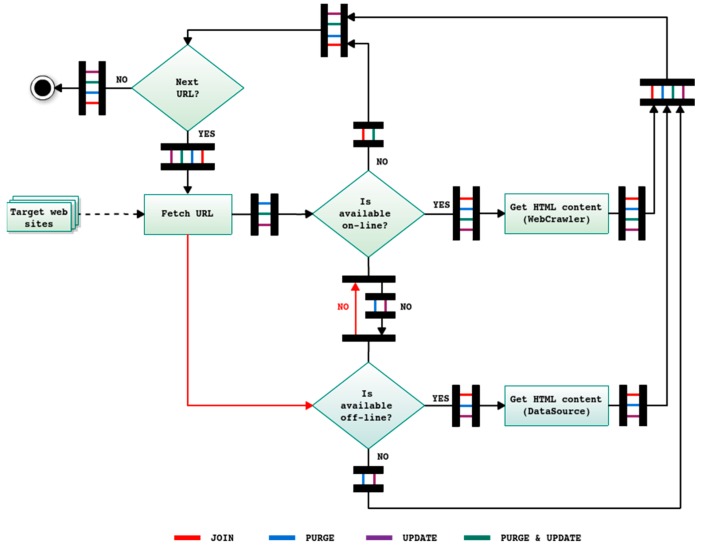
Detailed WARCProcessor operation mode for each task.

Depending on the specific task to be executed (from those represented in the central part of Figure 1), WARCProcessor carries out different actions when processing each URL (belonging to the list of the target web sites). Therefore, the two first phases of the workflow are common (i.e., independent of the specific task required by the user) whilst the third stage incorporates the particular logic implemented in WARCProcessor for each case (see Figure 2).

In detail, the join task specifically supports the compilation of contents from the user configured data sources. Under this scheme, the local content of each web site (existing WARC file) is always preferred than download it again (red line in Figure 2). This task intentionally excludes those entries initially included in the list of target web sites whose content is not accessible on Internet (online) or in (local) WARC files. The main advantage of this approach is the speed of the compilation process, which is increased as more web sites are locally available. In contrast, the purge task (blue line in Figure 2) does not include in the final corpus those web sites not available online, being the local copy of the HTML content always preferred for those existing URLs. For its part, the update task (purple line in Figure 2) is similar to a join task in which the online HTML content is preferred over the local version of the underlying URL. Finally, the purge and update task (green line in Figure 2) only selects those web sites that are available online, which have to be downloaded again.

### 3.2. System Architecture, Design and Implementation

**Figure 3:**
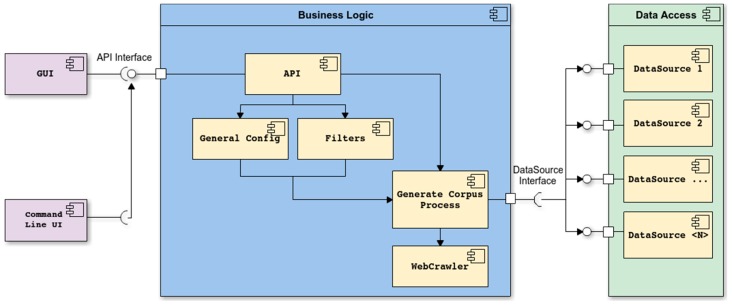
WARCProcessor modular architecture.

Since its inception, WARCProcessor was built following a straightforward modular architecture with the specific goal of enabling designers and programmers to easily extend the system with new functionalities. The layered architecture of WARCProcessor is introduced in Figure 3, where main dependencies between components are identified.

As shown in Figure 3 (from left to right), the architecture of WARCProcessor is divided into three main layers: user interface, business logic and data access. At the left side, the user interface implements both the GUI and the command line interface, which allows the unattended execution of the tool based on a configuration file. In the middle of Figure 3 is the business logic layer, which has two main responsibilities: (i) provide to previous layer all the required methods related to general input settings and the management of filters for URL processing and (ii) implement the required logic for running the web crawler and generate the output corpus. At the right side, the data access layer supplies the appropriate interface for reading and writing heterogeneous data sources, which might be subject to changes in the future to ensure scalability/adaptability. Additionally, Figure 3 also shows that all the actions coming from the user interface layer (GUI or command line) will use our specific Application Programming Interface (API) to interact with the different components comprising the business layer.

**Figure 4:**
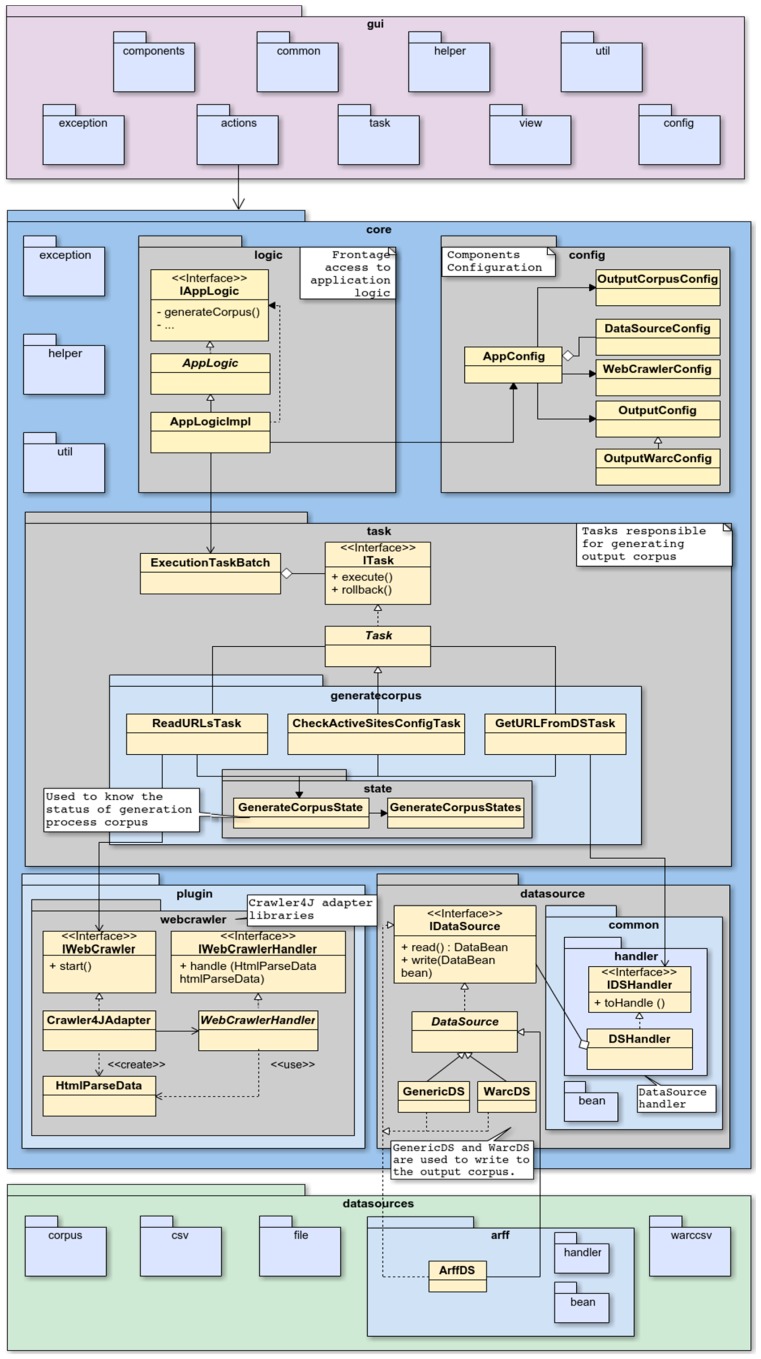
WARCProcessor package structure.

With the goal of providing a more in-depth view of WARCProcessor, Figure 4 introduces the package structure covering the three layers of the application. As it can be observed from Figure 4, WARCProcessor is formed by three main packages: gui, core and datasources. This natural division has as specific objectives to both increase cohesion and reduce coupling between the different elements that compose the application.

In order to guarantee a straightforward architecture, the GUI design follows the Model/View/Controller (MVC) structural pattern with the objective of decoupling the user interface from the business logic and data. Additionally, the model (represented by the core package) is accessed through an API providing the IappLogic interface, which defines the necessary methods to interact from the view (through class’s controllers, or actions) with the business logic of the application. In respect of the configuration package, it is divided into six different classes (each one related to a specific part of the application) with the goal of maintaining a high degree of cohesion. For its part, the Task package contains those classes in charge of the output corpus generation whereas the web crawler adapter is included in the plugin package. Additionally, the datasource package provides both basic classes and interfaces for handling supported data sources. Finally, the datasources package is designed with the purpose of providing a scalable architecture, giving specific support to the inclusion of novel input resources without changing the code. As a complement to the previously mentioned, Table 3 describes the specific contents and functionality of each package and sub package comprising our WARCProcessor application.

Concerning the implementation of WARCProcessor, it was coded in Java programming language (version 1.7) making use of Swing to implement the GUI. Additionally, in order to give support to different available functionalities, WARCProcessor integrates the following open source third–party libraries: Crawler4j (http://code.google.com/p/crawler4j/), Log4j (http://logging. apache.org/log4j/1.2/), Heritrix-common (http://sourceforge.net/projects/archive-crawler/), Warc Record (http://boston.lti.cs.cmu.edu/clueweb09/wiki/tikiindex.php?page=Working+with+ WARC+Files), Weka (http://weka.wikispaces.com/) and finally Apache Derby (http://db.apache.org/derby/).

In detail, the web crawler used in WARCProcessor was Crawler4j, which is an open source project distributed under the Apache License 2.0 providing a high degree of customization and versatility for crawling web content. For its part, Log4j implements an open source Java library that allows different levels of granularity for runtime data logging. It was mainly used in our code to centralize both information and error messages coming from different components through the same library (by configuring the log4j.xml file). Heritrix-commons was used in WARCProcessor to generate customized WARC files, whereas the WarcRecord library (belonging to the Lemur Project) was used just to parse WARC archives. Complementarily, our WARCProcessor tool also took advantage of Weka, a collection of machine learning algorithms written in Java that gives specific support to several data mining tasks. This library was exclusively used due to the ease of managing different file formats, such as CSV or ARFF. Finally, Apache Derby was selected as relational database for object persistence because it can be easily embedded into Java applications, it does not require installation and it is an open source under Apache License 2.0.

### 3.3. Execution Modes

From the user perspective, WARCProcessor supports two different but complementary modes of execution: the interactive GUI (for initial parameter definition and data source configuration) and its command line interface (for later background corpus generation). This section provides a brief overview about all the supporting functionalities available in both modes.

**Figure 5:**
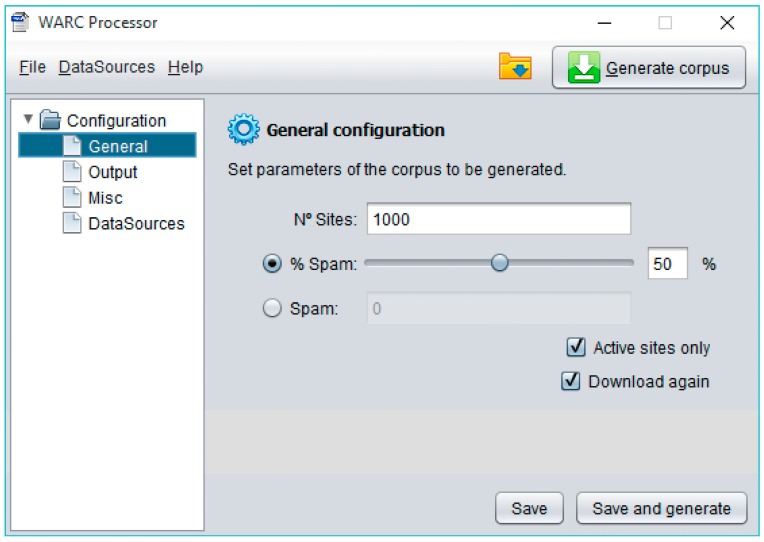
General configuration tab in WARCProcessor.

WARCProcessor organizes all the parameters governing the generation of a novel corpus as a straightforward configuration consisting of four main sections: General, Output, Miscellaneous and DataSources (see the left tree in Figure 5).

First of all, General configuration stores information concerning the characteristics of the target corpus, such as the maximum number of web sites that will make it up, the percentage (balance) of spam sites finally present vs. legitimate entries, as well as other information useful to correctly manage obsolete links (i.e., only considering active sites or forcing their final download). Secondly, Output configuration lets to specify the name of the folders used to generate the resulting files (allowing the elimination of their previous content). Later, the Miscellaneous configuration supports the specification of internal parameters governing the operation of WARCProcessor. These parameters include a path to a temporary folder, the number of threads to be used by the web crawler, the maximum deep for the site scanning operation and whether a redirection should be followed when it is initially found in the main site. Finally, DataSources configuration allows the specification of several available web spam corpora (containing already classified spam/legitimate sites) from different data sources. As previously commented, WARCProcessor currently supports up to six different types of data sources (i.e., csvDS, arffDS, fileDS, warcDS, warccsvDS and corpusDS).

**Figure 6:**
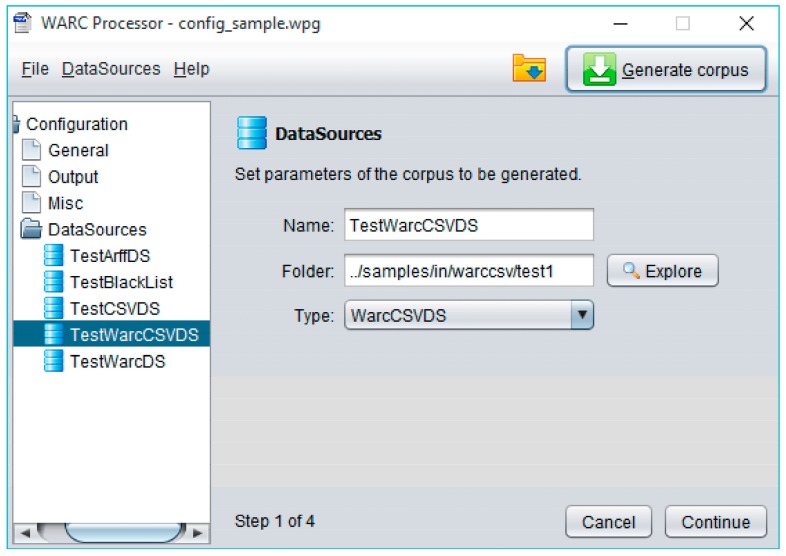
Specific data source configuration in WARCProcessor.

Every data source can be easily created in WARCProcessor by using the New option from the DataSource menu (located at the top of Figure 6). For each new data source being defined, WARCProcessor evolves over a 4-step straightforward wizard (showed in the right pane of Figure 6) that allows the establishment of a name (useful to further identify the original data source), a path to its corresponding data files and its respective type (from the six currently supported). Next steps comprising the wizard give access to complementary functionalities like the possibility of specifying the language(s) of the target corpus (filtering those entries not compatible with the user selection) as well as specifying configuration details of each specific data source.

**Figure 7:**
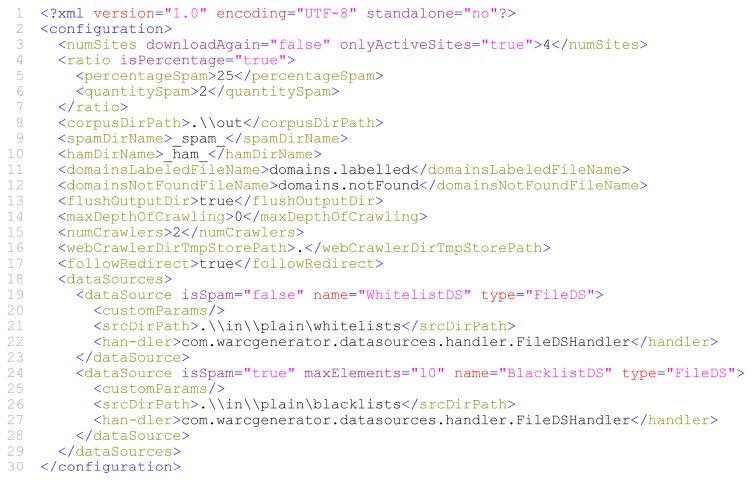
Snippet of a valid WARCProcessor configuration file.

As soon as the configuration process is finished, WARCProcessor save all the information to an editable .wpg file in XML format (see Figure 7) for later use.

In order to provide an overall view about the configuration possibilities directly supported by our tool, Table 4 summarizes all the available entities and attributes that can be used in a given XML WARCProcessor configuration file.

**Figure 8:**
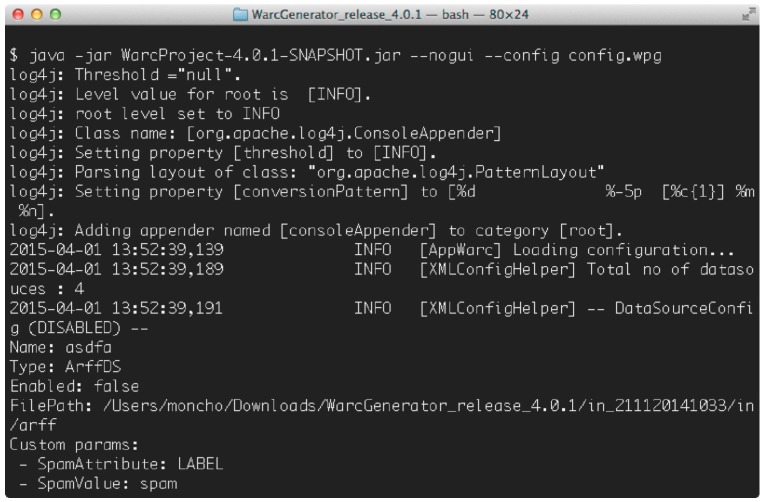
WARCProcessor running in batch mode.

Starting from a valid configuration file, the user can launch the generation of the new corpus (in WARC format) using either the GUI interface or the command line utility provided by WARCProcessor. If the graphical interface is used, as soon as the generation process finishes, the output folder containing the target corpus is automatically opened in the tool. Otherwise, the user needs to run WARCProcessor in batch mode using the flag –no–gui (see Figure 8).

## 4. System Evaluation

Given the fact that (to the best of our knowledge) there is not an equivalent tool designed with the same goal as WARCProcessor, there were no previously available metrics to compare with. In such a situation, and with the aim of evidencing the utility of the developed tool, we designed and executed a straightforward and reproducible benchmarking protocol that prove its effectiveness and efficiency in practice.

More specifically, in order to both facilitate the evaluation of future versions of WARCProcessor and enable other approaches to be compared with it, we have focused the evaluation procedure on computing the time spent for generating the output corpus under different circumstances. In detail, the most important procedures affecting the global performance of WARCProcessor are: (i) the initial retrieval of URLs from several input data sources stored in different formats (second phase in Figure 1) and (ii) the later compilation of their HTML content belonging to each target web site (third phase in Figure 1, zoomed in Figure 2).

In this line, all the experiments were executed on a computer running Windows 8 (64-bits) equipped with a two-core AMD 6-4400 APU 2.70 GHz processor and 6 GB of RAM. Complementarily, the time measured for each operation (in milliseconds) was averaged from the execution of three simulations using the same number of URLs sequentially read form each data source. For all the experiments, it was required to complete a list of target web sites comprising 1, 2, 3 … 10, 20, 30 … 100, 200, 300 … 1000, 2000, 3000 … 10,000 different URLs.

**Figure 9:**
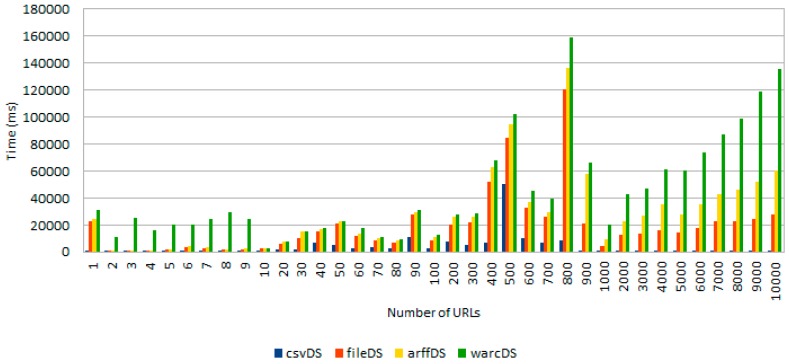
Time required to accomplish the initial phase comprising the retrieval of a variable number of URLs from different data sources.

The first experiment was designed with the goal of assessing the performance of the tool when processing a different number of URLs stored in four distinct data sources (i.e., csvDS, fileDS, arffDS and warcDS), each one containing 10,000 entries. Figure 9 shows the obtained results.

As Figure 9 demonstrates, there is a clear distinction between the average processing time needed to sequentially read the required amount of URLs from each data source. These differences are due to various implementation details of each specific data source as well as the required libraries used to read from different formats. From the information showed in Figure 9, it can be concluded that warcDS is the data source that requires a longer processing time, followed by arffDS, fileDS and csvDS. However, the completely processing time required for the initial retrieval of URLs (second phase in Figure 1) took 2.6 min in the worst case, something that is clearly manageable.

**Figure 10:**
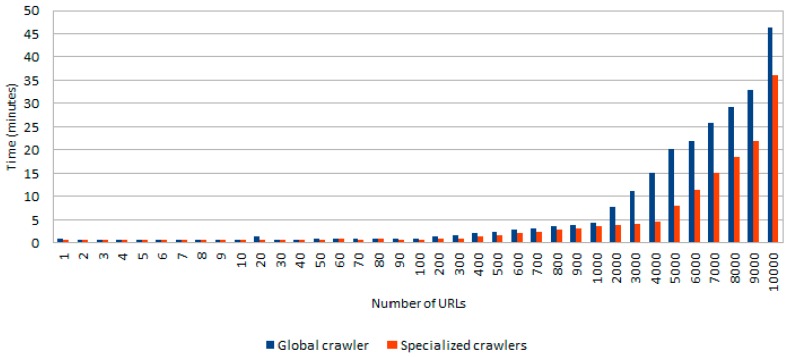
Time required to compile the HTML content of a variable number of URLs using one and two crawlers.

As previously mentioned, after the initial identification of target web sites WARCProcessor evolves by compiling their associated HTML content (see Figure 1). The particular details of the executed algorithm depends on the specific use case being supported (i.e., join, update, purge or purge & update corpus) but it is obvious that this phase is mainly affected by the particular web crawler implementation (Crawler4J in our case) and/or network latency. In this sense, and with the goal of standardizing the results of the experiment as much as possible we used WireMock (http://wiremock.org/), a flexible library for stubbing and mocking web services. The objective was to correctly simulate the response of a web server that is always up and running, being able to provide the HTML content of the required web sites. Additionally, we carried out the experiment under two different scenarios: (i) using a single (global) instance of the crawler in charge of processing all the target web sites and (ii) executing two (specific) instances of the crawler, one for downloading spam entries and the other for gathering legitimate sites. Figure 10 shows the average processing time (in minutes) for both scenarios varying the number of URLs that should be processed.

As we can observe from Figure 10, the total download time required for gathering the target content linearly grows with the number of URLs to be processed. In any case, a list containing up to 10,000 (spam and legitimate) web sites only requires 46 min for downloading all its associated HTML content when using a global crawler (blue bar in Figure 10), whereas with the exploitation of two specialized crawlers WARCProcessor achieves time-savings of up to 21.74% (only using 36 min for obtaining the same HTML content).

## 5. Conclusions and Further Work

Building a corpus is a tedious and error prone task requiring both great care and special skills for adequately collecting, structuring and analyzing existing data stored in different incompatible formats. With the aim of giving specific support to all the singularities that characterize research activities working with this type of information, in this work we present the design, implementation and evaluation of WARCProcessor, a platform-independent integrative tool providing specific support to scientists that need to perform experiments in the field of web spam research. The tool and the source code is freely available from the project homepage on http://sing-group.org/warcprocessor.

Although web sites can be closed or changed with the passage of time, the dynamism of WWW becomes especially relevant with the introduction of Web 2.0 trends (where changes may occur in seconds). In relation with the theoretical and practical implications of our research work for guaranteeing the correct verification of previous experimental findings, WARCProcessor allows storing local copies of compiled corpuses to correctly train and evaluate proposed approaches applied to the web spam domain. Local copies are stored using the WARC standard format together with complementary metadata files for the generation of novel corpora, which facilitates sharing data sets among the scientific community.

To the best of our knowledge, it is the first open source tool giving specific support to automate core functionalities which are essential for researchers working in this domain. Additionally to its modular design and flexible architecture, WARCProcessor provides two different but complementary execution modes: (i) a friendly interactive GUI designed for facilitating the initial configuration of different data sources and (ii) a command line interface for launching the corpus generation process in background.

The main current challenge for WARCProcessor is to be able to further reduce the time required for the generation of the target corpus, which actually involves a large number of I/O (slow) operations. In this line, it should be improved the parallelization of those tasks related with the internal management of the web crawler. Complementarily, we are also implementing novel algorithms to directly support the execution of feature extraction procedures over the target corpus with the goal of automatically generating .csv and arff files.

Finally, due to both the growing popularity experimented by online social networks and other Web 2.0 platforms, together with their fraudulent usage to distribute spam contents, we believe there is a real need to implement complementary tools to compile comprehensive datasets. These corpus should compile relevant information including user profiles, tags, comments, and other Web 2.0 elements required for measuring the performance and benchmarking available and novel techniques in a reproducible way. Hence, these developments will be another important challenge in the near future.

## Figures and Tables

**Table 1:** Available corpora for web spam research.

Collection Name		Hosts	Pages	Domain Crawled	Language	Spam Labelled Hosts (%)	Format	Publicly Available
uk	2006 ^1^	11,402	77.9M	United Kingdom (.uk)	English	26%	warc	Y
	2007 ^2^	114,529	105M			5.3%		Y
	2011 ^3^	n/a	3767			53%		Y
DC	2010 ^4^	99,000	23M	Europe (.eu)	English French German	3.2%	warc	Y
Webb spam	2006	n/a	350,000	links found in millions of spam e-mails	English	100%	arff	Y
	2011 ^5^		330,000					Y
clueweb	09 ^6^	20M	500M	United States, United Kingdom, Canada, Australia, Ireland, New Zealand	English	4.95%	warc	N
	12 ^7^	33,447	733M			0.07%		N

^1^ http://chato.cl/webspam/datasets/uk2006/; ^2^ http://chato.cl/webspam/datasets/uk2007/; ^3^ https://sites.google.com/site/heiderawahsheh/home/web-spam-2011-datasets/uk-2011-web-spam-dataset; ^4^ https://dms.sztaki.hu/en/letoltes/ecmlpkdd-2010-discovery-challenge-data-set; ^5^ http://www.cc.gatech.edu/projects/doi/WebbSpamCorpus.html; ^6^ http://www.lemurproject.org/clueweb09.php/; ^7^ http://www.lemurproject.org/clueweb12.php.

**Table 2:** Available data sources natively supported by WARCProcessor.

Data Source	Description
csvDS	It is composed of one or more .csv (Comma Separated Values) files in compliance with RFC 4180, where each row contains information about a given web site. All the files included in the data source should have the same number of attributes.
	Although .csv files can theoretically define values for an unlimited set of features, WARCProcessor only takes advantage of two specific web site attributes: the URL (required) and its associated class (optional if all the entries belongs to the same class: spam or legitimate).
arffDS	It encapsulates one or more ARFF files (http://www.cs.waikato.ac.nz/ml/weka/arff.html) describing a list of web sites characterized by certain common attributes. As in the case of csvDS, WARCProcessor only takes into consideration the site URL (required) and the instance class (optional). Therefore, the configuration of this data source is analogue to csvDS.
fileDS	It contains one or more ASCII text files (.txt) storing each URL as a single line without additional information. This type of data source can only be used to specify web sites belonging to a unique class (spam or legitimate). The specific class of the data source is defined through a configuration parameter when it is created in WARCProcessor.
warcDS	It consists of one or more standard WARC (Web ARChive) files (http://www.iso.org/iso/iso_catalogue/catalogue_tc/catalogue_detail.htm?csnumber=44717) storing the content of previously downloaded web sites belonging to a single class (spam or legitimate). According to the general configuration of our WARCProcessor tool, the HTML content of the web sites included in this data source can be either directly used to generate the output corpus or downloaded again (if they are still available).
warccsvDS	It combines the advantages of both warcDS data source (able to store the HTML content of a web site) and csvDS (able to specify the URL of the web site and its associated class). Therefore, this data source provides the same functionality as csvDS with the additional advantage of avoiding the need of downloading again all the web sites.
corpusDS	It support a complementary way of storing into a single data source the whole HTML contents of a set of sites, their URLs and the corresponding classes. As long as the HTML content and the URL of a given web site can be also stored into a WARC file, this data source uses different system folders to separate WARC files containing spam and legitimate entries. Given its simplicity and flexibility, this data source is used by WARCProcessor to store the output corpus.

**Table 3:** Functionality provided by each package in WARCProcessor.

Main Package	Sub Package	Description
gui	.actions	Contains all event handlers related with interface controls.
	.common	Set of classes commonly used by other application components.
	.components	Custom Swing components adding new features.
	.config	Correspond to the configuration of the GUI.
	.exception	Set of exceptions that can be thrown by the GUI.
	.helper	Set of classes to delegate tasks and improve cohesion.
	.task	Set of classes representing tasks, which require a greater degree of control over their execution and must be completely executed to successfully finalize an activity.
	.util	Help libraries.
	.view	Swing components that compose the GUI.
core	.config	Related with the system configuration. It is divided in several classes for managing the specific configuration of the several elements that compose the application.
	.datasource	Contains the main sources of information used for read/write operations.
	.exception	Set of exceptions that can be thrown by the application.
	.helper	Set of classes to delegate tasks and improve cohesion.
	.logic	Business application logic.
	.plugin	Adapters for external classes. In the current version, a specific adapter for Crawler4J libraries is available.
	.task	Responsible for the generation of the output corpus.
	.util	Help libraries.
datasources	.corpus	Abstraction layer based on input and output streams that encapsulates the logic for obtaining and writing data from/to different sources.
	.csv	
	.file	
	.arff	
	.warccsv	

**Table 4:** Description of XML elements supporting any .wpg WARCProcessor file.

Configuration Context	XML Element	Description
General	numSites	Stands for the maximum number of websites to be included in the corpus. This element also contains the mandatory attributes *downloadAgain* and *onlyActiveSites* used to configure the operation when an obsolete link is found.
	ratio	If the attribute *isPercentage* is true, the *percentageSpam* element will be interpreted as the percentage of spam websites required for the output corpus. Otherwise, *quantitySpam* element determines the number of spam sites that will be included in the output.
Output	corpusDirPath	Indicates the output directory.
	spamDirName	Indicates the output subdirectory where information classified as spam will be stored.
	hamDirName	Indicates the output subdirectory where information classified as ham will be stored.
	domainsLabeledFileName	Specifies the name of the file where pages that could be read and classified will be written.
	domainsNotFoundFileName	Specifies the name of file where pages that could not be processed will be written.
	flushOutputDir	Indicates if the output directory should be emptied before building the new corpus.
Other	maxDepthOfCrawling	Defines the maximum depth search for the web crawler.
	numCrawlers	Specifies the number of crawlers to be used when tracking the URLs.
	webCrawlerDirTmpStorePath	Defines a temporary directory to be used by the web crawler to store processing information.
	followRedirect	Indicates if a site redirection should be followed.
Data source	datasource	Contains three attributes (i.e., *name*, *type* and *isSpam*). The data source name is included in *name* whilst *type* indicates the kind of data source (i.e., *CSVDS*, *FileDs*, *CorpusDS*, *WarcDs*, *ArffDS*). *isSpam* (optional) determines whether all websites included in the data source will be treated as spam or legitimate. If this attribute is not set, each website should be individually labeled.
	maxElements	Defines the maximum number of URLs that will be obtained from a data source (optional).
	customParams	Specifies the collection of attributes required for each kind of data source (optional).
	srcDirPath	Indicates the directory containing the files of the data source.
	handler	Specifies the class used to process information before being tracked by the web crawler. It can also be used to modify the output file.
CSV data source	spamCol	Specifies the name or column number that indicates whether the URL is spam or ham. If this element is empty, *spamColSpamValue* is not taken into account.
	spamColSpamValue	Identifies the column (established by *spamCol*) that indicates if the URL contains a spam site. Any other value will be considered as ham.
	fieldSeparator	Specifies the character acting as field separator in the CSV file.
	urlCol	Specifies the name or column number containing the URL of the target website.
File data source	n/a	
Corpus data source	spamDir	Indicates the subdirectory containing spam files.
	hamDir	Indicates the subdirectory containing ham files.
	urlTag	Specifies the name of the tag included in the header of each WARC record stores the URL of the site to be indexed.
Warc data source	urlTag	Specifies the name of the tag (included in the header of each WARC record) that stores the URL of the site to be indexed.
Arff data source	urlTag	Specifies the column name containing the URL to be downloaded.
	spamTag	Indicates which column is used to know if the URL is spam.
